# Remedia Sternutatoria over the Centuries: TRP Mediation

**DOI:** 10.3390/molecules26061627

**Published:** 2021-03-15

**Authors:** Lujain Aloum, Eman Alefishat, Janah Shaya, Georg A. Petroianu

**Affiliations:** 1 Department of Pharmacology, College of Medicine and Health Sciences, Khalifa University of Science and Technology, Abu Dhabi 127788, United Arab Emirates; lujain.aloum@ku.ac.ae (L.A.); Eman.alefishat@ku.ac.ae (E.A.); 2 Center for Biotechnology, Khalifa University of Science and Technology, Abu Dhabi 127788, United Arab Emirates; 3 Department of Biopharmaceutics and Clinical Pharmacy, Faculty of Pharmacy, The University of Jordan, Amman 11941, Jordan; 4 Pre-Medicine Bridge Program, College of Medicine and Health Sciences, Khalifa University of Science and Technology, Abu Dhabi 127788, United Arab Emirates; shaya.janah@ku.ac.ae

**Keywords:** sternutation, transient receptor potential (TRP), sneezing, agonist

## Abstract

Sneezing (sternutatio) is a poorly understood polysynaptic physiologic reflex phenomenon. Sneezing has exerted a strange fascination on humans throughout history, and induced sneezing was widely used by physicians for therapeutic purposes, on the assumption that sneezing eliminates noxious factors from the body, mainly from the head. The present contribution examines the various mixtures used for inducing sneezes (remedia sternutatoria) over the centuries. The majority of the constituents of the sneeze-inducing remedies are modulators of transient receptor potential (TRP) channels. The TRP channel superfamily consists of large heterogeneous groups of channels that play numerous physiological roles such as thermosensation, chemosensation, osmosensation and mechanosensation. Sneezing is associated with the activation of the wasabi receptor, (TRPA1), typical ligand is allyl isothiocyanate and the hot chili pepper receptor, (TRPV1), typical agonist is capsaicin, in the vagal sensory nerve terminals, activated by noxious stimulants.

## 1. Introduction

When we sneeze (sternutatio), one of the upper airway reflexes, starting with a deep inspiration proceeded by involuntary expulsion of air [1]. The reflex is mediated by the vagal afferent input from the nasal receptors [2,3]. Transient receptor potential (TRP) channels have been reported to play a role in sneezing as nasal receptors [4]. The TRP channel superfamily consists of 28-cation channels divided into six subfamilies. The TRPA1 (ankyrin subfamily, member 1) is known as the wasabi receptor (typical ligand is allyl isothiocyanate, the main pungent compound of wasabi) and the TRPV1 (vanilloid subfamily, member 1) is known as the hot chili pepper receptor (typical ligand is capsaicin, the pungent compound of hot chili pepper) [5,6,7,8]. TRPA1 and TRPV1 have been associated with sneezing [4].

Subsequent to the excitation of receptors in the nasal mucous membranes, sensory information is transmitted mainly via the nasopalatine nerve (branch of the maxillary nerve; NC V2), while accompanying parasympathetic afferent fibers run to the pterygopalatine ganglion, and then to the superior salivatory nucleus. A putative sneezing center in the medulla is activated and a complex motor pattern is initiated: a deep inspiration is triggered, accompanied by the head tilting to the back, eye closure and nasopharyngeal and glottis closure. The airway closure allows for the intrathoracic pressure to build up following contraction of the expiratory muscles, which is followed by head tilting to the front, glottis opening and an explosive expulsion of air from the lungs (hopefully of the noxious stimulus) [9,10].

Sneezing had great significance and value throughout history; it exerted a strange fascination on humans. Despite the fact that, during the Middle Ages, sneezing was a threat due to the spread of the Plague disease, sneezing was generally seen as a positive reflex throughout history [11]. Induced sneezing was widely used by physicians for therapeutic purposes, on the assumption that sneezing eliminates noxious factors from the body, mainly from the head. Sneezing protects the upper airway and efficiently clears the nose and nasopharynx [1]. The Greek philosophers from the fourth century BC depicted sneezing as a divine sign. Hippocrates said that sneezing is helpful to diseases, except in the setting of pulmonary illnesses. In Pagan culture, sneezing was considered to expel the devil from the human body. Furthermore, Celsus considered sneezing an important part of disease recovery [11]. The Romans and Greeks depicted sneezing as an indication of well-being [10].

Physicians over the centuries utilized intranasal powders/mixtures to induce sneezing. In this review, we explored various mixtures and their constituents, used as remedia sternutatoria (or sneeze-inducing) over the centuries, aiming to conclude the mechanism of action via which these remedies trigger sneezing.

## 2. Remedia Sternutatoria: Terminology

The various sneeze-inducing powders are named Errhina, Ptarmica or Sternutatoria (remedia). Errhina (into the nose) are substances which, if snuffed up the nose, promote a discharge of mucus from that organ, and can induce occasional sneezing. The name Ptarmica comes from the Greek word ptairo (to sneeze) and means “causes sneezing”, as such, it is a synonym of the Latin sternutatoria, or a remedy that excites sneezing [12]. Parr (1819) stated that very little difference appears between the different articles that constitute this class, with the exception of their strength. Betony, sweet marjoram, the orris root, and rosemary tops are of the milder kind (Errhina), while asarum, euphorbium, tobacco, white hellebore, and the turbith mineral are of the stronger kind (Remedia sternutatoria) [13].

## 3. Remedia Sternutatoria: Main Active Components

Table 1 lists the various herbs and natural remedies used by numerous physicians over the centuries to induce sneezing.

*Cumini* or *Cuminum cyminum* (cumin) is a popular hot spice in many parts of the world; the ancient Greeks apparently kept cumin at the dining table in its own container, and this practice continues in parts of North Africa. In medieval Europe, cumin was used as a cheap(er) substitute for black pepper [28]. Legrand and his colleagues found that cuminal, the main active ingredient of cumin [29], specifically activates TRPA1 channels without affecting TRPV1 [30].

*Piperis* or *Piper nigrum* (black pepper), the source plant of both black and white pepper, contains the main active constituent alkaloid piperine [31,32]. Piperine activates TRPV1 [33,34], where it was found to bind to the same binding pocket as capsaicin [35]. Capsaicin (a vanilloid), the active component found in chili powder (*Capsicum*) and cayenne pepper, is the typical agonist of TRPV1 [36]. Dong et al. revealed that threonine 671 (T671) found on the pore-forming S6 segment is a vital site for the piperine-induced activation of TRPV1. It is assumed that piperine directly interacts with the S6 segment to facilitate the opening of the channel [35]. Furthermore, the piperidine ring, proposed to be important for TRPV1 activity as its replacement with *N*,*N*-diisobutyl, eliminated TRPV1 interactions [37]. Other studies found that piperine and its analogs activate not only TRPV1, but also TRPA1 [38,39]. The half-maximal effective concentration (EC50) of piperine for TRPV1 and TRPA1 was found to be 250 and 58 times higher than capsaicin and allyl isothiocyanate (a typical agonist of TRPA1), respectively [38].

**Figure 1:**
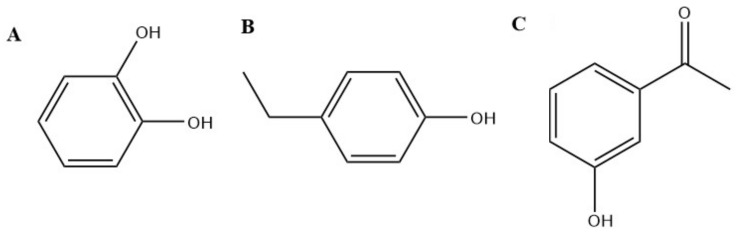
Chemical structures of (**A**) catechol, (**B**) 4-ethylphenol and (**C**) 3-hydroxyacetophenone.

*Herba castorii*, despite its name (herba; wort), is not a plant, but rather a castoreum (Bibergeil), the yellowish exudate from the castor sacs of the mature beaver [40]. In particular, 1,2-dihydroxybenzene (catechol) (Figure 1A), 4-ethylphenol (Figure 1B), acetophenone and 3-hydroxyacetophenone (Figure 1C) are four compounds with the strongest pheromonal activity in castoreum [41]. It was found that catechol estrogens positively regulated TRPA1 activity [42]. Acetophenone was found to be a weak agonist of TRPA1 [43].

Some *Euphorbio* plants (specifically *Euphorbia resinifera*) contain resiniferatoxin, a (3-4 orders of magnitude) more potent, functional analog of capsaicin. It causes severe burning pain in sub-microgram quantities when ingested orally [44]. Resiniferatoxin has been used in several studies for its ability to activate TRPV1 [45,46,47].

**Figure 2:**
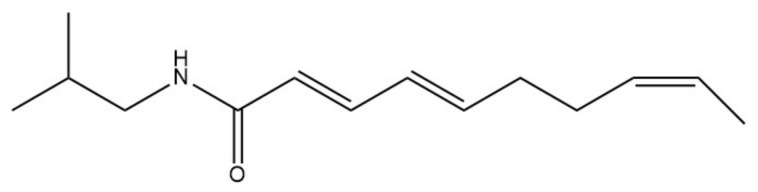
Chemical structure of 8,9-*Z*-dehydropellitorine.

*Achillea ptarmica*: the name ptarrmica comes from the Greek word ptairo (sneeze), while achillea is related to the hero’s heel and the belief that the plant can heal wounds. The colloquial Italian name of the plant is sternutella, while in English it is called sneezewort. It is assumed that alkyl-amides are responsible for most pharmacological actions of the *Achillea ptarmica* [48]. Pellitorine (a trans isomer) and 8,9-*Z*-dehydropellitorine (Figure 2) are the main alkamides of *Achillea ptarmica* [48,49]. Trans-Pellitorine, an aliphatic alkylamide analogue of capsaicin, is reported to activate TRPV1 and TRPA1 channels; however, recent studies have found that this is mediated indirectly, possibly via the downstream signaling of an unknown molecular target [50,51]. On the contrary, Pellitorine, extracted from *Tetradium daniellii*, was found to be an antagonist at the TRPV1 channel [52].

*Nicotianae* species are recognized as tobacco plants. Snuff is made from finely ground tobacco leaves. When applied locally, nicotine often causes a sneeze [53]. The main alkaloid in tobacco is nicotine [54]. Nicotine demonstrated a bimodal effect on TRPA1 channels, in which low and high concentrations resulted in the activation and inhibition of the channel, respectively [55,56], similar to the effect at nicotinic cholinergic receptors [57]. Notably, Talavera showed that the TRPA1 channel regulates the nicotine-induced nasal irritating effect rather than nicotinic cholinergic receptors [55]. Furthermore, the TRPV1 response to capsaicin was found to be sensitized by nicotine [58]; however, a later study found that nicotine-inhibited TRPV1 [55].

**Figure 3:**
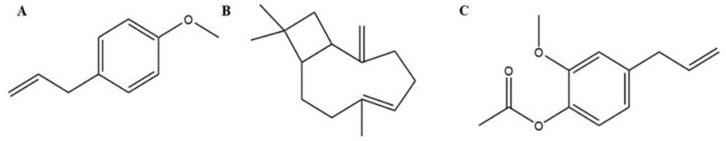
Chemical structures of (**A**) methyl chavicol, (**B**) beta-caryophyllene and (**C**) eugenyl acetate.

*Ocimum basilicum* oil comprises eugenol and methyl chavicol (Figure 3A) as the main constituents [59]. Eugenol, beta-caryophyllene (Figure 3B) and eugenyl acetate (Figure 3C) are the chief active ingredients in *Caryophylli flos*, recognized as cloves [60]. It was found that eugenol activates TRPA1 [61,62,63] and TRPV1 [64,65].

*Sinapis alba* (white or yellow mustard) and *Sinapis nigra* (black mustard) both contain the active component, allyl isothiocyanate, which is one of the main ingredients accountable for the mustard taste [66,67]. Allyl isothiocyanate is produced when the sinapis plant is processed (e.g., crushed), allowing myrosinase isoenzymes to convert sinalbin and sinigrin, in *Sinapis alba* and *Sinapis nigra*, respectively, into allyl isothiocyanate [66,68]. Allyl isothiocyanate constitutes approximately 70% of the mustard seed essential oil [69]. Not only is allyl isocyanate the typical agonist of TRPA1 [61,70], but also it exerts an inhibitory effect on TRPA1 at high concentrations (millimolar), suggesting bimodal activity [71]. Furthermore, this compound was recently found to activate TRPV1 channels with approximately 10–100-fold higher concentrations compared to capsaicin [71,72,73]. Mustard oil is considered a partial agonist of TRPV1. Furthermore, it was shown that mustard oil causes fast desensitization of TRPA1, but not TRPV1 [71].

*Urtica dioica* L. is commonly recognized as “stinging nettle”. The main constituent of its essential oil is carvacrol [74]. Carvacol is reported to activate and desensitize TRPA1 [75,76,77,78].

**Figure 4:**
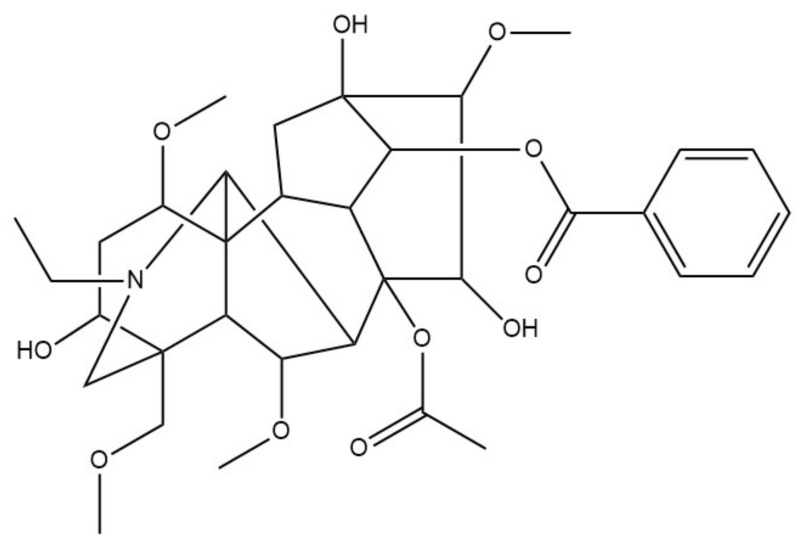
Chemical structure of aconitine.

*Ranunculus* contains the main active component aconitine (Figure 4). Aconitine has been shown to antagonize TRPV1 channels, exerting mixed competitive and non-competitive allosteric regulation [79].

Cardamom (*Elettaria cardamomum*) contains 1,8-cineole as one of the main active constituents [80]. This bicyclic monoterpenoid has been shown to activate TRPA1, but not TRPV1 channels [81]. In contrast, Takaishi and his colleagues reported that 1,8-cineole is a natural antagonist of human TRPA1 channels [82]. This bimodal action has been reported with menthol, in which the activity at the mouse TRPA1 was concentration dependent; it was an agonist at low concentrations and an antagonist at higher concentrations [83].

*Aframomum melegueta*, commonly known as grain of paradise or guinea grains, contains rich amounts of 6-paradol, 6-gingerol, 6-shogaol and other pungent compounds. The main components of ginger extract are 6-gingerol and 6-shogaol [84]. Other studies found that 6-paradol is also one of the main components [85]. These compounds have been shown to activate TRPV1 [86,87,88] and TRPA1 [61,88,89].

**Figure 5:**
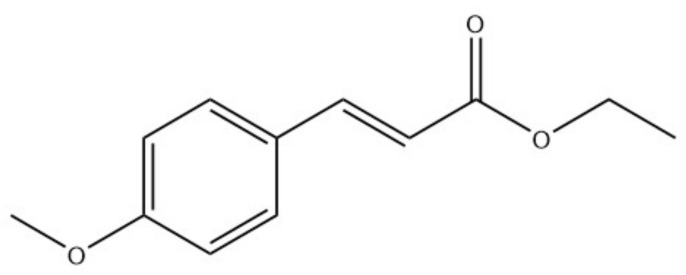
Chemical structure of ethyl trans-*p*-methoxycinnamate.

Galangal, reported over history, is most probably in reference to either greater galangal (*Alpinia galanga*) or lesser galangal (*Alpinia officinarum*) or black galangal (*Kaempferia galangal*). The key pungent compound of *Alpinia galanga*, 1′-acetoxychavicol acetate, was found to activate TRPA1 in HEK cells; it expressed those receptors, but did not activate TRPV1. Notably, 1′-acetoxychavicol acetate showed higher potency (3.8-fold lower EC50) than the usual TRPA1 agonist (allyl isothiocyanate) [90]. Several compounds have been isolated from *Alpinia officinarum*; galangal appears to be the principal component in various parts of the plant, such as the leaves, rhizomes and aerial parts [91,92]. Galangal was found to activate TRPA1 at low micromolar concentrations without affecting TRPV1 [92]. Several major components of the essential oil of *Kaempferia galangal* have been reported with ethyl trans-*p*-methoxycinnamate (most abundant, Figure 5), 1,8-cineole, etc. [93]. The latter was found to activate TRPA1 channels [81].

Coriander (*Coriandrum sativum*) essential oil composition differs in proportion depending on environmental conditions. However, linalool was consistently found to be the most abundant constituent [94,95], which activates TRPA1 without affecting TRPV1 [88,96].

**Figure 6:**
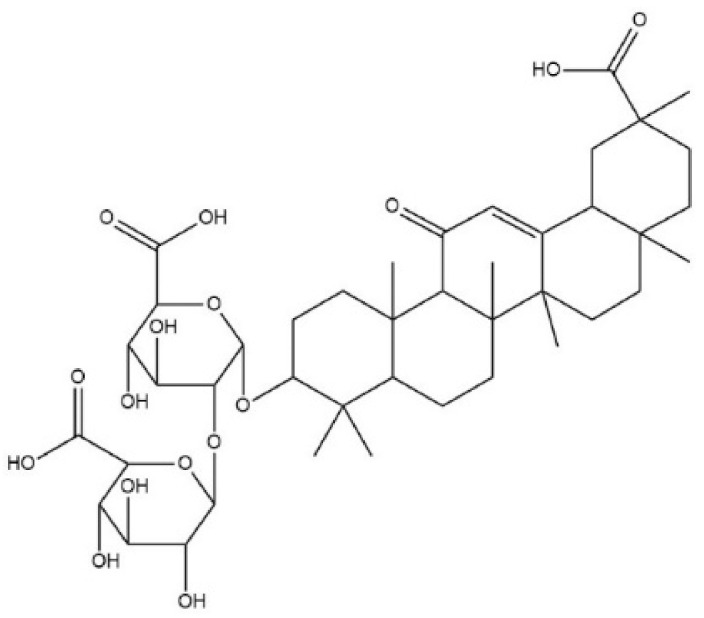
Chemical structure of glycyrrhizin.

The roots of liquorice (*Glycyrrhiza glabra*) contain the chief active component glycyrrhizin, (Figure 6) which can be hydrolyzed to glycyrrhetinic acid in the human body [97]. The latter was found to activate TRPA1 [98].

**Figure 7:**
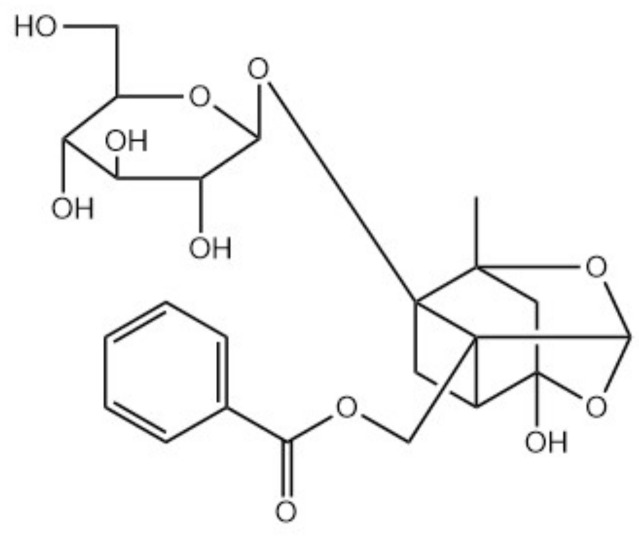
Chemical structure of paeoniflorin.

*Paeoniae* (genus) includes paeoniflorin (monoterpene glycoside) as the main active component (Figure 7) [99,100]. It has been reported to exert inhibitory activity against TRPV1 expression and TRPV1-mediated effects [101].

Myrrh contains volatile oil, resin, gum and a bitter component [102]. Curzerene is the most abundant constituent of the essential oil obtained from the oleo-gum resin of *Commiphora myrrha* [103]. The water extract of frankincense and myrrh was found to inhibit TRPV1 [104].

Camphor is a volatile, terpenoid ketone. It can either be obtained from the wood of the camphor tree (*Cinnamomum camphora*) or it can be chemically synthesized from turpentine [105]. Camphor was found to activate and desensitize TRPV1 channels [106]. Notably, it is reported that camphor exhibits a bimodal concentration-dependent effect on TRPA1 channels: at high concentrations it blocked the channel [106,107], while at low concentrations it activated the receptor [107].

*Asarum* (genus) is commonly known as wild ginger due to its rhizomes and ginger root similarity in taste and smell. Most of the plants belonging to the genus *Asarum* contain aristolochic acid [108]. Aristolochic acid might activate TRPA1 channels as TRPA1 antagonists suppress the thermally dependent taste effect of aristolochic acid [109]. In addition, methyl eugenol was found to be the most abundant constituent of the essential oils of the majority of *Asarum* species [108]. It activated human TRPA1, exhibiting higher EC50 (~160 μM) than allyl isothiocyanate (EC50 ~ 7 μM) [110].

**Figure 8:**
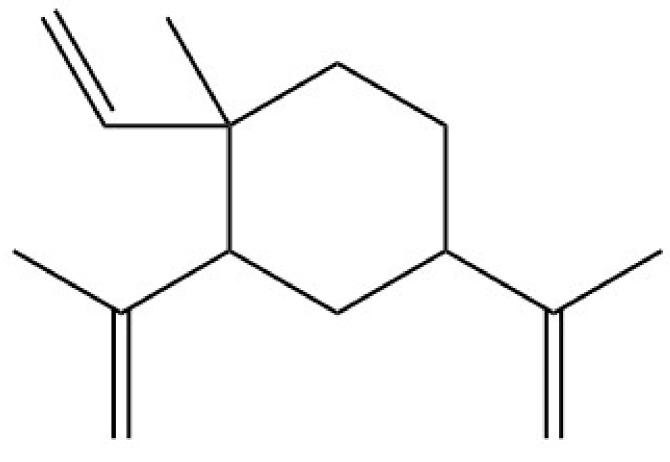
Chemical structure of β-elemene.

*Nigella damascena* is an annual plant; its seeds are characterized by a strawberry flavor and unique aroma [111]. β-elemene (Figure 8) was found to be one of the main components in the essential oil of *N. damascena* seeds and one of the compounds responsible for its unique odor [111,112,113]. Computational studies predicted that β-elemene can activate TRPA1 [114].

*Cubebarum*, known as tailed pepper, is mainly grown for its fruits and essential oil [115]. Many papers studied its chemical composition. Methyleugenol, eugenol, sabinene, eucalyptol, 4-terpineol, β-pinene, camphor, elemene, α-copaene, β-caryophyllene, epi-cubebol, cubebol and δ-3-carene were reported as the main constituents isolated from the essential oils of the fruits of *P. cubeba* [115,116,117,118]). Eugenol, camphor and methyl eugenol were shown to be activators of TRPA1 [61,62,63,107,110], while the two latter compounds were found to activate TRPV1 [64,65,106].

## 4. Discussion

TRP channels play numerous physiological roles, such as thermosensation, chemosensation, mechanosensation and osmosensation [119,120,121,122,123,124]. They are linked to several pathophysiological conditions, such as pain, cardiac hypertrophy, respiratory reflex hypersensitivity, cancer and genetic diseases [4,125,126,127,128,129,130]. For example, TRP melastatin 7 (TRPM7) channels has been shown to affect the viability of breast cancer cells via modulation of the cell cycle [126,127]. Furthermore, TRP canonical (TRPC) channels upregulation is involved in cardiac hypertrophy; its activation results in an increase in calcium. TRPC plays an important part in the regulation of the signaling cascades involved in cardiac hypertrophy [125,128]. TRPA1 and TRPV1 channels have well-established roles in pain and neurogenic inflammation [131,132]. In addition, non-neuronal TRPA1 and TRPV1 play a role in cardiovascular disease, immunity and other conditions [133]. Remarkably, it has been reported that almost all sensory neurons expressing TRPA1 (approximately 97%) express TRPV1 [134]. Functional cross-desensitization has also been reported between the typical agonists of TRPA1 (allyl isothiocyanate) and TRPV1 (capsaicin) [135]. Furthermore, studies have shown that TRPA1 and TRPV1 can form a complex in the plasma membrane, and therefore influence each other’s characteristics [136]. Thus, Fernandes et al. described TRPA1 and TRPV1 channels as “partners in crime” [133].

The mammalian respiratory tract is highly innervated with sensory fibers expressing TRPA1 and TRPV1. Sneezing occurs because of the activation of these sensory fibers (expressing TRPA1 and TRPV1) in the vagal sensory nerve terminals by noxious stimulants [4]. Signal transduction occurs by augmenting intracellular calcium concentrations and/or depolarizing membrane potentials [137,138]. It was found that there is an overexpression of TRPV1 in the nasal mucosa and augmented substance P levels in nasal secretions in idiopathic rhinitis patients [139].

**Figure 9:**
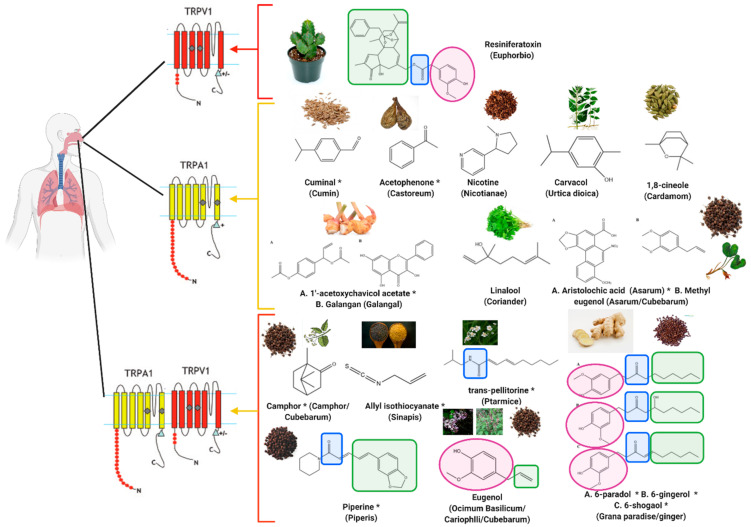
Agonists of TRPA1 and TRPV1 reported to induce sneezing over centuries (pink shape indicates vanillyl group, blue shape indicates polar entity (instead of amide group), green shape indicates aliphatic group); * represents electrophilic compounds activating TRPA1 via the reported or suggested covalent modification of the cysteine residues of the intracellular *N*-terminal. Generated by Biorender.com (accessed on 15 February 2021).

Despite the fact that that only a few mixtures contained antagonists of TRPA1 and TRPV1 (which probably induced sneezing because of mechanical stimulation of the nasal receptors [1]), the majority of the constituents of the sneeze-inducing mixtures, used historically by physicians, are activators of TRPV1 and TRPA1 (Table 2). Figure 9 summarizes the structures of the constituents of the sneeze-inducing agents reported to activate TRPA1 and TRPV1. The big array of reported agonists supports the fact that these channels function as polymodal detectors [140,141].

TRPA1 and TRPV1 channels have six transmembrane (S1–S6) polypeptide subunits, permeable to cations via a pore formed by a tetramer assembly [142]. The hydrophobic pore region is located between S5 and S6 [143]. TRPA1 and TRPV1 contain ankyrin repeat motifs at the intracellular *N*-terminal; however, TRPA1 possess a remarkably high number of ankyrins [4]. TRPV1 and TRPA1 agonists both interact with binding sites to activate the channels.

TRPA1 has a very distinct mechanism of activation. Agonists activate TRPA1 via covalent modifications of the cysteine and lysine residues located in the ankyrin repeat motifs within the cytoplasmic *N*-terminus of the channel. Thus, TRPA1 activators can be categorized into thiol-reactive electrophilic compounds, which activate TRPA1 via covalent modification, and non-electrophilic compounds that activate TRPA1 via ligand binding [144,145]. Allyl isothiocyanate, an electrophile, was shown to form adduct with thiols and primary amines, proposing covalent modification. Notably, it was reported that thiol-reactive compounds of various structures activated TRPA1 via this mechanism [146]. Cuminal, acetophenone, acetoxychavicol, aristocholic acid, piperine, 6-paradol, 6-gingerol, 6-shogaol, pellitorine and camphor all potentially activate TRPA1 via covalent modification of the cysteine/lysine residues because of their electrophilic characteristics. This supports the finding that most of the noxious stimuli and electrophilic compounds activate TRPA1 via this distinctive mechanism, which explains its polymodal activity [122,147]. However, nicotine and carvacol have been shown to activate TRPA1 via non-covalent interaction with the channel [55,78]. The plausible mechanism of TRPA1 activation by linalool, 1,8-cineole, eugenol and methyl eugenol is via a similar non-covalent mechanism, as they are weak or non-electrophilic molecules.

**Figure 10:**
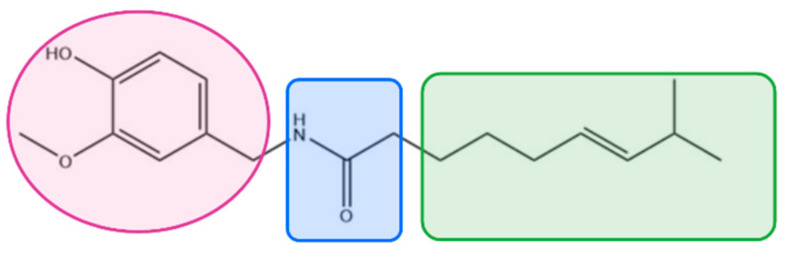
Chemical structure of capsaicin, a typical TRPV1 agonist (pink shape indicates vanillyl group, blue shape indicates amide group, green shape indicates aliphatic group).

Yang et al., 2015. showed that the aliphatic chain of capsaicin (Figure 10, a typical agonist of TRPV1) forms nonspecific Van der Waals interactions with the channel, contributing to binding affinity. On the other hand, the amide and vanillyl groups’ interactions with T551 and E571, respectively, via hydrogen bonding, are essential for the specificity of ligand binding [148]. Resinferatoxin comprises a very similar pharmacophore to capsaicin, including vanillyl ester (similar to the amide group) and aliphatic groups, explaining its activity against only TRPV1. However, eugenol, 6-paradol, 6-gingerol and 6-shogaol maintained the vanillyl group, but not the amide, which might explain the additional activity against TRPA1. In the majority of these compounds, the amide group was replaced with polar entities and it was reported that this type of replacement reserves functionality, justifying their TRPV1 activity [149]. On the contrary, the lack of the vanillyl group in methyl eugenol led to a loss of TRPV1 activity reported with eugenol. Essentially, Del Prete et al. revealed an orthogonal structure–activity relationship for TRPV1 and TRPA1 binding, and proposed that agonists acting on both TRPA1 (non-electrophilic) and TRPV1 can be obtained by modifying the capsaicin pharmacophore [150].

Several binding sites have been reported for TRPV1 agonists. This might explain the reported TRPV1-inducing activity of camphor and allyl isothiocyanate, which lack the capsaicin pharmacophore. After interaction with residues in the S4–S5 linkers, capsaicin and resiniferatoxin twitch them away, thus opening the channel. It was found that the pore region is extremely flexible, and thus, every agonist might result in a different gating mechanism [143].

## 5. Conclusions

Historically, physicians commonly used various mixtures to induce sneezing; however, they were unaware and lacking knowledge of the molecular mechanism that explains this reflex. Today, after examining the constituents of these sneeze-inducing remedies, the majority are found to be agonists of TRPA1 and TRPV1 channels. This is in parallel with the role of TRPA1 and TRPV1 channels in sneezing. The paper sheds light on the potential area of research on TRP antagonism to treat sneezing. Additional investigations are required to explore the structure–activity relationship of these active ingredients.

## Figures and Tables

**Table 1:** Herbs and natural remedies used by various physicians over the centuries to induce sneezing.

Name as Reported over History	Current Name	Physicians															
		Pedanius (~40–90)	Aëtius (≈†574)	De Gordon (1270–1330)	De Montagna (1400–1460)	Fuchs (1501–1566)	Dalla Croce (1509–1575)	Joel (1510–1579)	Joubert (1529–1582)	Zacuth (1575–1642)	Chalmeteus (~ 1560:)	Schoock (1614–1669)	Woyt (1671–1709)	Andrioli (1672–1713)	Arnemann (1763–1806)	Par (1750–1810)	Hesselbach (1788–1856)
Cumini	*Cuminum cyminum*		**X**			**X**											
Piperis	*Piper nigrum*		**X**	**X**	**X**	**X**	**X**	**X**	**X**	**X**	**X**	**X**					
Herba castorii/castoreum	Castoreum	**X**	**X**	**X**		**X**	**X**	**X**		**X**		**X**	**X**				
Euphorbio	*Euphorbia resinifera*					**X**	**X**	**X**	**X**		**X**	**X**	**X**			**X**	
Ptarmice	*Achillea ptarmica*	**X**				**X**						**X**					
Nicotianae	Tobacco leaves									**X**			**X**	**X**	**X**	**X**	
*Ocimum basilicum*	*Ocimum basilicum*	**X**															
Cariophlli	Caryophylli flos											**X**					
Sinapis		**X**										**X**					
Urtica	*Urtica dioica*				**X**												
Ranunculo		**X**															
Cardamom	*Elettaria cardamomum*								**X**								
Aframomum-Grana paradise	*Aframomum melegueta*																
Ginger	*Zingiber officinale*				**X**				**X**								
Galangal					**X**				**X**								
Coriander	*Coriandrum sativum*												**X**				
Salt liquorice-Salzlakritz	*Glycyrrhiza glabra*												**X**				
Paeoniae										**X**							
Myrrhae	*Commiphora myrrha*									**X**							
Kampfer	Camphor														**X**		
Asarum																**X**	**X**
*Nigella damascena*	*Nigella damascena*												**X**				
Cubebarum	*Piper Cubeba*								**X**								
Cyclamini			**X**			**X**											
Elleboro albo	*Veratrum album*			**X**	**X**	**X**	**X**	**X**	**X**	**X**	**X**	**X**	**X**	**X**	**X**	**X**	**X**
Daphnoides-Spurge Laurel	*Daphne laureola*	**X**															
Pyrethri							**X**				**X**	**X**		**X**			
Mercurius dulcis	Hydrargyrum chloratum dulce														**X**		
Chinapuder	China powder														**X**		
Nitro	Saltpeter					**X**											
Aloe										**X**					**X**		
Herba lanaria	*Saponaria officinalis*	**X**	**X**			**X**											
Staphydis agriae	*Staphysagria macrosperma*				**X**						**X**						

Pedanius Dioscorides (c. 40–90 AD) was a Greek physician, pharmacologist, botanist, and author of a multivolume encyclopedia of herbal medicine [14]. Aëtius of Amida (≈† 574) was a Greek physician at the court of Byzantine Emperor Justinian [15]. Gordon, Bernard de (1270–1330) was a French physician teacher; his books, specifically *Lilium medicinae*, formed an important part of the medical curriculum of medieval Europe [16]. De Montagna, Bartholomaeus (ca. 1400–ca. 1460) was an Italian physician and professor in Padua and Bologna. Fuchs, Leonhard (1501–1566) was a German physician and botanist; professor of medicine and Chancellor of the University of Tübingen in Württemberg, Germany [17,18]. Della Croce, Giovanni Andrea (1509–1575) was an Italian surgeon who authored “Universal Surgery Complete with All the Relevant Parts for the Optimum Surgeon”, which was a keystone for traumatology [18]. Joel, Franciscus primus (Joó Ferenc; 1510–1579) was a Hungarian-born pharmacist and physician, professor, and then, the rector of the University in Greifswald, Duchy of Pomerania [19]. Joubert, Laurent (1529–1582) was the Chancellor of the Faculty of Medicine at the University of Montpellier [20]. Zacuth, Abraham (1575–1642) was famous for his precise descriptions of diseases, such as the plague and blackwater fever [21]. Chalmeteus, Antonius (Antoine Chaumette) was a French surgeon who authored “Enchiridion chirurgicum, externorum morborum remedia complectens” [22]. Schoock, Martin (1614–1669) was a Dutch historian and professor at the University of Deventer and the University of Frankfurt (Oder) [23]. Woyt, Johann Jacob (1671–1709) was a German physician and professor of medicine at the University of Königsberg (Prussian city that is now Kaliningrad, Russia) [24]. Andrioli, Michele Angelo (1672–1713) was an Italian physician who worked in Klagenfurt, Austria. He was a prolific author who assisted Barbato, Girolamo in the discovery of blood serum. Arnemann, Justus (1763–1806) was a German surgeon and professor of medicine at the University of Göttingen [25]. Parr, Bartholomew (1750–1810) was a British surgeon and Fellow of the Royal Society of Edinburgh and London [26]. Hesselbach, Adam Kaspar (1788–1856) was a German professor of surgery in Würzburg and Bamberg. He was the son of Franz Kaspar Hesselbach, a famous surgeon known for his contribution to hernia surgeries [27].

**Table 2:** The constituents of sneeze-inducing remedies and their TRP modulatory activity.

Sneeze-Inducing Remedies	Main Component	TRP Channel Agonist
Cumini	Cuminaldehyde	TRPA1
Piperis	Piperine	TRPV1 and TRPA1
Castoreum	Acetophenone	TRPA1
Euphorbio	Resiniferatoxin	TRPV1
Ptarmice	trans-Pellitorine *	TRPV1 and TRPA1 (indirect)
Nicotianae	Nicotine **	TRPA1
*Ocimum basilicum*/Cariophlli/ Cubebarum	Eugenol	TRPA1 and TRPV1
Sinapis	Allyl isothiocyanate **	TRPA1 and TRPV1
*Urtica dioica*	Carvacol	TRPA1
Cardamom	1,8-cineole **	TRPA1
Aframomum-Grana paradise/ Ginger	6-paradol	TRPV1 and TRPA1
	6-gingerol	TRPV1 and TRPA1
	6-shogaol	TRPV1 and TRPA1
Galangal	1′-acetoxychavicol acetate	TRPA1
	Galangan	TRPA1
Coriander	Linalool	TRPA1
Salt liquorice-Salzlakritz	Glycyrrhizin → glycyrrhetininc acid	TRPA1
Camphor/Cubebarum	Camphor **	TRPA1 and TRPV1
Asarum	Aristolochic acid	TRPA1
Asarum/Cubebarum	Methyl eugenol	TRPA1

* Pellitorine (Ptarmice), TRPV1 antagonist; ** TRPA1 antagonist, Nicotine (Nicotianae); Paeoniflorin (Paeoniae) and Aconitine (Ranunculus), TRPV1 antagonists. → indicates hydrolysis of glycyrrhizin to glycyrrhetinic acid in human body.
